# *Announcement*: National Birth Defects Prevention Month and Folic Acid Awareness Week — January 2018

**DOI:** 10.15585/mmwr.mm665152a6

**Published:** 2018-01-05

The Zika virus outbreak and response led to renewed focus on how infections can increase the risk for having a baby born with a birth defect. “Prevent Infections for Baby’s Protection” is the theme of January 2018’s National Birth Defects Prevention Month. Birth defects are common, costly, and critical conditions that affect one in 33 U.S. babies annually (*1*). Not all birth defects can be prevented, but women can increase their chances of having a healthy baby by reducing their risk for getting infections before and during pregnancy.

Women who are pregnant or might become pregnant can take the following steps to prevent infections: talk to their health care provider about how they can reduce their risk for infections; get vaccinated to help protect against influenza (*2*) and pertussis (*3*); protect themselves from insects, such as mosquitoes, known to carry infections, including Zika (*4*); and reduce contact with saliva and urine from babies and young children to prevent infections such as cytomegalovirus (*5*). CDC encourages everyone to join this nationwide effort to raise awareness of birth defects, their causes, and their impact. Additional information is available at https://www.cdc.gov/ncbddd/birthdefects/prevention-month.html.

January 7–13, 2018 is National Folic Acid Awareness Week. CDC urges all reproductive-aged women to get 400 *µg *of folic acid every day to help reduce the risk for serious birth defects of the brain and spinal cord (spina bifida and other neural tube defects) (*6*). Women can get folic acid from fortified foods, supplements, or both. This guidance is especially important for Hispanic/Latina women, because this group has the highest rate of pregnancies affected by neural tube defects and the lowest reported consumption of folic acid (*7*). Additional information about folic acid is available at https://www.cdc.gov/ncbddd/folicacid/index.html. 
